# Inhibition of Pseudomonas aeruginosa Biofilm Formation Using Silver Nanoparticles

**DOI:** 10.7759/cureus.77848

**Published:** 2025-01-22

**Authors:** Suvarna Yadav, Satyajeet Pawar, Satish Patil

**Affiliations:** 1 Department of Microbiology, Krishna Institute of Medical Sciences, Krishna Vishwa Vidyapeeth, Karad, IND

**Keywords:** antibiofilm assay, biofilm inhibition, biofilm production, pseudomonas aeruginosa, silver nanoparticles, tissue culture plate assay

## Abstract

Background and aim

Nanotechnology explores the unique properties of nanoparticles, which are very dissimilar from their bulk forms. Silver (Ag) has been exhibited to have antimicrobial and antibiofilm properties; since ancient times, silver has been used for its therapeutic qualities. Currently, medical research is investigating the activity and potential uses of silver nanoparticles (AgNPs). *Pseudomonas aeruginosa* is frequently seen in nosocomial settings because of its capacity to form biofilms on medical devices, implants, and instruments, which increases the risk of infection in hospitalized patients. When *P. aeruginosa* forms biofilms, it becomes more resistant to antimicrobials and can persist on medical equipment. Biofilms contribute to drug resistance and can drive the progression from acute to chronic diseases. Novel approaches can be aimed at a co-treatment strategy that mixes a drug that disrupts biofilms with conventional antibiotics, and this may make the biofilms more susceptible to treatment. In the present investigation, we study the antibiofilm effect of AgNPs against the biofilm production of *P. aeruginosa*.

Materials and methods

The study included 196 *P. aeruginosa* isolates from specimens at Krishna Hospital & Medical Research Center, Karad. Identification (ID) and antibiotic susceptibility testing (AST) were done by using the VITEK 2 compact system (BioMérieux, France). Biofilm production and antibiofilm assays were evaluated using the tissue culture plate (TCP) method.

Results

Biofilm production was observed in 171 (87.24%) of isolates, with 25 (12.76%) being non-biofilm producers. Among biofilm producers, 91 (46.43%) were weak, 58 (29.59%) moderate, and 22 (11.22%) strong. At 400 µg/mL of AgNP concentration, 68 (85%) of isolates showed 60%-90% inhibition.

Conclusions

*P. aeruginosa* is a significant hospital-associated pathogen, as indicated by its isolation rate of 16.15%, emphasizing its clinical importance. Notably, 87% of isolates were biofilm producers. Nanotechnology, particularly AgNPs, presents promising solutions for combating biofilms, offering versatile and effective approaches for healthcare and industrial applications.

## Introduction

Nanotechnology is an interdisciplinary field of study that focuses on the diverse characteristics of nanoparticles (NPs). The physicochemical properties of NPs differ greatly from those of their bulk counterparts. NPs ranging from 1 to 100 nm exhibit special and unusual characteristics [1]. Silver has been demonstrated to have antimicrobial activity against a variety of bacteria. It has been used for medicinal purposes since ancient times. Currently, medical research is investigating the activity and potential uses of silver NPs (AgNPs) [2].

AgNPs’ increased antibacterial activity at the nanoscale has proven particularly useful in the medical and healthcare sectors, such as surgical and food handling instruments, apparel, cosmetics, dental products, catheters, and dressings. AgNPs’ diverse modes of action, which allow them to kill a variety of bacteria by attacking several structures at once, contribute to their potential as antibiotics [3].

Among the most well-known metallic NPs for contemporary antibacterial applications is AgNPs [4]. According to several studies, AgNPs engage with the bacterial membrane and enter the cell, causing structural damage, cell death, and a severe disruption in normal cell function [5]. Many scientists have investigated the bactericidal activity of AgNPs against pathogenic, multidrug-resistant (MDR), and susceptible strains of bacteria. It has been accepted that AgNPs are effective weapons against MDR bacteria, including *Pseudomonas aeruginosa*, methicillin-resistant *Staphylococcus aureus* (MRSA), vancomycin-resistant *S. aureus* (VRSA), erythromycin-resistant *Streptococcus pyogenes*, and ampicillin-resistant *Escherichia coli* [6].

*P. aeruginosa* is a rod-shaped, motile, non-fermenting, pigment-producing gram-negative aerobic bacteria. Due to its ubiquitous nature, this organism can be isolated from various environmental settings. In immunocompromised people, it is among the most prevalent opportunistic pathogens, and patients are admitted to the intensive care unit (ICU) [7]. It can be colonized in patients from various sources, e.g., medical devices such as ventilators, surgical devices, and biomaterials like catheters, prostheses, and contact lenses, as well as sanitary and cleaning equipment like sinks, hot tubs, and showers [8]. It does not need much nutrition to survive and can endure a range of environmental conditions. These are the main reasons for nosocomial infections, and all help opportunistic microorganisms succeed [9,10]. It may live for six hours to six months in a hospital setting on dry, inert/inactive surfaces because of its high degree of adaptability and ability to survive in disinfectants [11]. A nosocomial infection is a healthcare-associated infection (HAI) that does not incubate upon admission and manifests at least 48-72 hours after admission or up to three days following discharge [12]. Ten percent of HAIs are caused by this pathogen, which ranks as the fourth most frequent isolated nosocomial pathogen [13]. Beyond its capacity to colonize biotic and abiotic surfaces, *P. aeruginosa* is frequently seen in nosocomial settings due to its potential to form biofilms on medical devices, implants, and instruments, which increases the threat of infection in hospitalized patients [14]. *P. aeruginosa* also contributes to an increase in patient morbidity and mortality rates. The potential to develop distinct pathways of antibiotic resistance allows bacteria to resist multiple antibiotics through different mechanisms, making them MDR and harder to treat [15]. The proportion of MDR *P. aeruginosa* isolates significantly increased in evaluation to the number of *P. aeruginosa* isolates in 2019 and 2020, potentially due to the COVID-19 era's longer hospital stays and higher antibiotic use [16].

Complicated communities of bacteria known as biofilms form on various surfaces and are surrounded by an extracellular polymeric substance (EPS) matrix that the organisms themselves manufacture. They are common and can grow on a variety of surfaces, causing biofouling and rusting, in industrial systems, and infections in healthcare facilities and medical devices. Due to biofilms, *P. aeruginosa* infection is one of the biggest challenges [17]. The complex structure of biofilms increases the pathogenic potential of these microbes, immune evasion, and persistent infections, making treatment challenging and prone to failure [18].

When *P. aeruginosa* forms biofilms, it becomes more antibiotic-resistant and can persist on medical equipment. This can result in chronic infections in individuals with cystic fibrosis, mechanical ventilators, and burn wounds [19]. A biofilm is made up of a self-secreted matrix consisting of water (97%) and proteins (<2%), DNA (<1%), polysaccharides (<2%), and RNA (<1%). The three exopolysaccharides that make up the predominance of the components in the biofilm matrix are alginate, psl, and pel. They perform a variety of biological tasks, particularly those related to defending the bacterial cell from antibiotics and the immune system of humans [20]. The genes expressed by biofilm cells differ from those of free-floating (planktonic) cells, which are mostly responsible for the development of antibiotic resistance in bacteria. They can create biofilms on both inert surfaces, like medical equipment, and living tissue, like wounds, to help them elude the host's immune system and withstand antibiotics. They cause around 80% of microbial infections and allow virulent strains to propagate by horizontal gene transfer. Regarding the treatment of MDR *P. aeruginosa* infections, AgNPs may have a major role in inhibiting the formation of biofilms [21]. Generally, biofilms are known to provide resistance against several antimicrobial agents, and biofilms can be the leading cause of a shift from acute-phase diseases to chronic diseases. New therapeutic strategies can be aimed at co-treatment strategies that mix a drug that disrupts biofilms with conventional antibiotics, and this may make the biofilms more susceptible to treatment. In this present investigation, we study the antibiofilm effect of AgNPs against the biofilm production of *P. aeruginosa*.

## Materials and methods

Study design

An observational prospective study is a type of research design where investigators observe and collect data about participants over a period of time without intervening or manipulating any variables. As there was no intervention in vivo, the study was observational prospective.

Study settings

As the study was carried out from specimens of hospital patients and carried out at the lab in the microbiology department, we have mentioned the same: Krishna Hospital and Medical Research Center, Karad, Department of Microbiology, Krishna Institute of Medical Sciences (KIMS), Krishna Vishwa Vidyapeeth, Karad.

Inclusion criteria

Non-repetitive, consecutive, clinical isolates of *P. aeruginosa* were included in the study.

Methodology

Over the study period, a total of 196 *P. aeruginosa* isolates obtained from Krishna Hospital and Medical Research Center, Karad, from various specimens, different wards, both sexes, and all ages for routine culture and sensitivity in the Department of Microbiology, KIMS, were included in this study and started after approval from the institutional ethics committee. Antibiogram, biofilm production, and inhibition by AgNPs of these isolates were investigated and recorded.

Isolation and identification of the strains

All microbiological specimens received during the period of one year for culture and sensitivity were performed in accordance with standard methodology. Specimens were sputum, pus, urine, endotracheal secretions, blood, body fluids (such as peritoneal fluid, pleural fluid, and ascitic fluid), and other specimens like catheter tips, except stool specimens. Specimens obtained after an invasive procedure and from the indwelling catheters were also included in the study. Specimens other than blood were cultured on MacConkey’s agar, blood agar, and chocolate agar (HiMedia, Thane, India) as per standard methods [22].

All these plates were incubated overnight at 37°C, and all the bacteria grown in these culture media were further processed for identification (ID) and antibiotic susceptibility testing (AST). *P. aeruginosa* was primarily screened by its colony characteristics, non-lactose fermentation, pigment production, grape-like odor, motility, gram-negative characteristic, and oxidase positivity. *P. aeruginosa* colonies were further processed and confirmed by the automated bacterial VITEK 2 compact system (BioMérieux, France) using a gram-negative ID card, and AST was done with the same system to detect minimum inhibitory concentration (MIC). VITEK 2 system ID and AST of bacteria are based on and are compliant with the Clinical and Laboratory Standard Institute (CLSI) guidelines 2021 [23,24].

Characterization of biofilm production

All clinical isolates were examined for biofilm production using the tissue culture plate (TCP) method: The TCP assay, which was initially described by Christensen et al. in 1985 [25], is considered the most acceptable method of determining biofilms. Biofilm formation was accomplished using a modified TCP test. After being cultivated in fresh agar plates for 24 hours, the isolates were re-inoculated in 5 mL of trypticase soy broth (TSB) containing 1% glucose and an adjusted 0.5 McFarland turbidity standard and incubated for a 24-hour incubation period at 37°C. Each well of the polystyrene 96-well flat-bottomed plate was filled with 180 µL of TSB containing 1% glucose and 20 µL of the culture (1:10 dilution using fresh media) on sterile TCPs. Sterile broth served as a negative control (background absorbance). After a 24-hour incubation period at 37°C, gentle tapping was performed. To remove free-floating cells, 200 μL of phosphate buffer (pH 7.2) was used, and four such washings were performed. Sessile or adherent bacteria created a biofilm, fixed by incubating the plate at 55°C for an hour and staining it with 200 µL of 0.1% crystal violet for 30 minutes. After washing the plates three times in tap water to remove any remaining stains, they were left to dry. After dissolving the stained adherent bacteria in 95% ethanol for 30 minutes, the optical density (OD) at 630 nm [26] was measured using a microplate reader (LisaQuant-TS, Tulip Diagnostics, India).

The cutoff value for the samples was calculated using the OD of the negative control:

ODc = average OD of negative control + 3 x SD of negative control

For every strain, the assays were run in triplicate, and a cutoff value was established each time. The cutoff value (ODc) was three standard deviations (SDs) above the mean of the OD of the negative controls. ODc is the average of the OD of the negative control plus three times the SD of the negative control. The findings were interpreted using Kamali et al.'s criteria [27]. Four classes have been created from the isolates:

• If OD of isolates < ODc = non-biofilm producer

• If ODc < OD of isolates < 2 × ODc = weak biofilm producer

• If 2 × ODc < OD of isolates < 4 × ODc = moderate biofilm producer

• If 4 × ODc < OD of isolates = strong biofilm producer

Antibiofilm assay

In order to make AgNP suspensions, 0.4 and 0.2 mg of powder, obtained from Amnium Technologies Private Limited in Pune, Maharashtra, with a particle size of 10-20 nm, were added to sterile tubes containing 1 and 2 mL of sterile double-distilled water, respectively. The particles were then dispersed thoroughly using a sonicator machine for 15 minutes. Serial dilutions were made in the tubes with concentrations of 12.5, 25, 50, 100, 200, and 400 μg/mL, starting with the initial concentration of AgNPs (400 and 100 μg/mL). An additional 15 minutes was spent sonicating each dilution. In the following step, *P. aeruginosa* isolates that were categorized as moderate and strong biofilm-producing were selected, and the bacterial suspension was prepared at 0.5 McFarland.

This method was conducted in 96-well microtiter plates (sterile, flat-bottomed 96-well plate with a lid), 180 μL of TSB, 10 μL of an overnight-grown culture whose turbidity was adjusted to match 0.5 McFarland standards, and 10 μL of AgNPs (at different concentrations: 12.5, 25, 50, 100, 200, and 400 μg/mL) added in each well. The experiment also included positive control wells without AgNP additions and negative control wells without bacterial suspensions. After being incubated for 24 hours at 37°C, the wells were carefully removed, and to take out the planktonic and unbound cells, phosphate buffer solution (pH 7) was used. Following three times of washing with 200 μL of phosphate-buffered saline (PBS), the biofilms were fixed using heat, and the incubation of microtiter plates was done for one hour at 55°C. To visualize the adhered cells, following fixation, the biofilms were stained using 200 μL crystal violet dye (0.1%) for 30 minutes. Following staining, the plates were left to dry before any remaining dye was thoroughly cleaned out of the wells using distilled water. The colored biofilm cells were eluted by adding 200 μL of ethanol (95%) to each well after they had dried. A volume of 125 μL of the ethanol solution was then transferred into a fresh microtiter plate after 30 minutes of incubation. A microplate enzyme-linked immunosorbent assay (ELISA) reader (LisaQuant-TS by Tulip Diagnostics) was used to assess the eluted cells' absorbance at 630 nm, which made it possible to quantify the cell number that may form biofilms. The average was determined by utilizing information from a minimum of three distinct biological replicates [28,29].

To calculate the percentage of biofilm inhibition, the following formula was used:

Biofilm inhibition (%) = (1 − absorbance of treated sample/absorbance of non-treated control sample) × 100 [30]

This calculation compares the absorbance of the treated samples (with AgNPs) against the absorbance of the control sample (without AgNPs), providing a measure of the effectiveness of the AgNPs in inhibiting biofilm formation.

Statistical analysis

The data collected was tabulated in MS Excel (Microsoft Corp., Redmond, WA). The analysis was incorporated by using Statistical Package for the Social Sciences (SPSS) version 28.0 (IBM, India Software). Normality was tested by the Shapiro-Wilcoxon test. The frequency and percentage of study variables were determined by using frequency distribution. The functional relationship between study variables as per the data matrix was done by using the chi-squared test.

## Results

This study included 196 *P. aeruginosa* isolates from different clinical specimens. The isolation rate of *P. aeruginosa* infection in the hospital was 16.15% out of 1,213 positive cultures and age-wise and gender-wise distribution as per Table 1.

**Table 1:** Age- and gender-wise distribution of Pseudomonas aeruginosa isolates.

Age group in years	Female	Percentage	Male	Percentage	Total	Percentage
<1-15	1	0.51	2	1.02	3	1.53
16-30	15	7.65	24	12.24	39	19.90
31-45	15	7.65	42	21.43	57	29.08
46-60	17	8.67	34	17.35	51	26.02
61-75	11	5.61	27	13.78	38	19.39
76-90	2	1.02	6	3.06	8	4.08
Total	61	31.12	135	68.88	196	100.00

**Figure 1:**
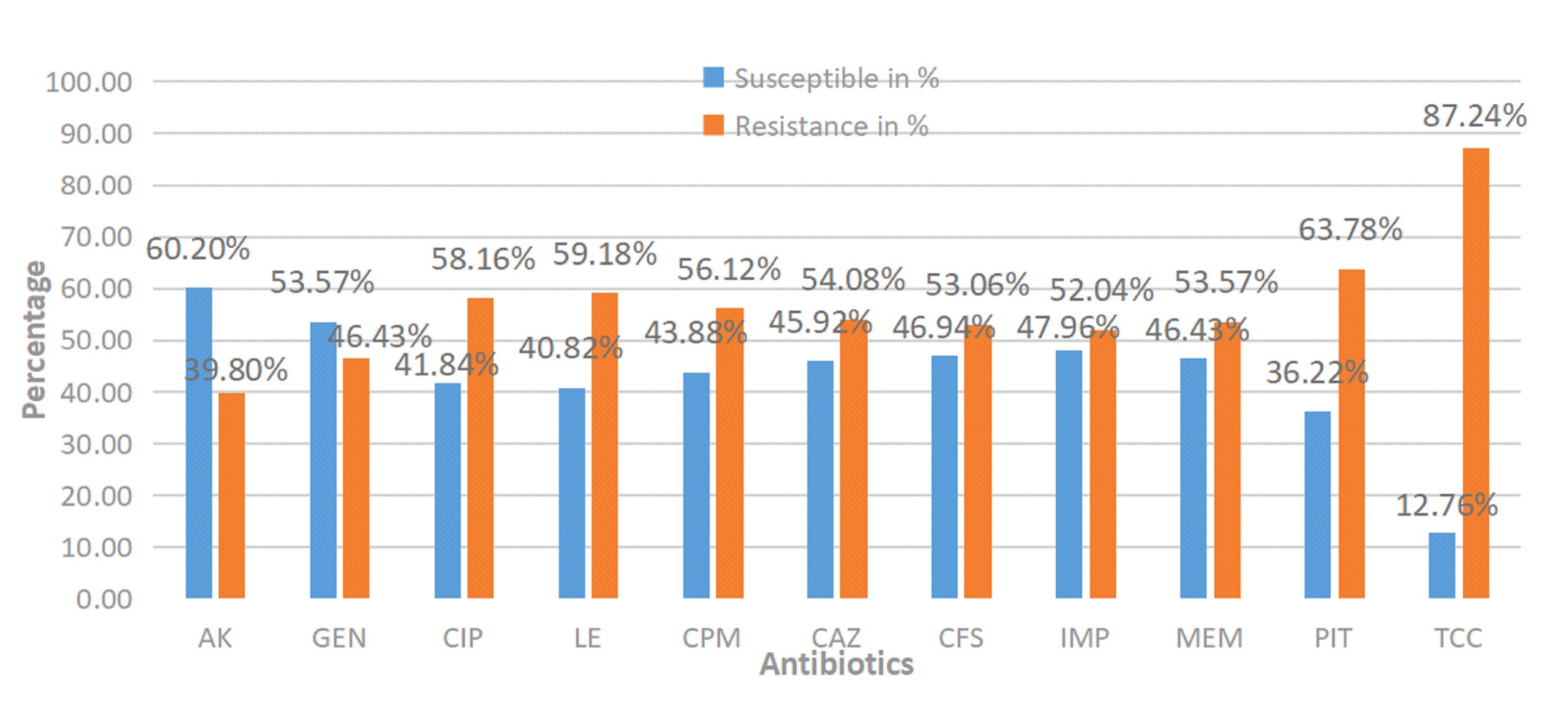
Antimicrobial susceptibility pattern of Pseudomonas aeruginosa clinical isolates. AK: amikacin; GEN: gentamicin; CIP: ciprofloxacin; LE: levofloxacin; CPM: cefepime; CAZ: ceftazidime; CFS: cefoperazone/sulbactam; IMP: imipenem; MEM: meropenem; PIT: piperacillin/tazobactam; TCC: ticarcillin/clavulanic acid

In this study, infections with *P. aeruginosa* were more common in men (135 (69%)) than women (61 (31%)). Most isolates were from the male age group 31-45 (42 (21.43%)), followed by the patient age group 46-60 (34 (17.35%)), with the least isolates from the female age group <1-15 (1 (0.51%)). The antibiograms of the collected isolates are given in Figure 1.

Maximum resistance was shown among combination drugs, ticarcillin/clavulanic acid (171 (87.24%)), piperacillin/tazobactam (125 (63.78%)), and cefoperazone/sulbactam (104 (53.06%)). Among the fluoroquinolones, maximum resistance was observed for levofloxacin (116 (59.18%)) and ciprofloxacin (114 (58.16%)), followed by cefepime (110 (56.12%)).

**Figure 2:**
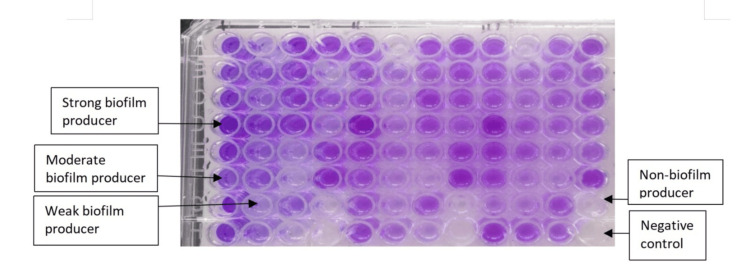
Biofilm production by tissue culture plate assay.

Total biofilm formation was observed in 171 (87.24%) of isolates. These biofilm producers were grouped into strong, moderate, weak, and non-biofilm producers as per Figure 2. 

Non-biofilm producers were 25 (12.76%). The high occurrence of the biofilm producer category was weak biofilm producers (91 (46.43%)), followed by moderate biofilm producers (58 (29.59%)) and strong biofilm producers (22 (11.22%)), as shown in Table 2.

**Table 2:** Biofilm production pattern among Pseudomonas aeruginosa (n = 196).

Biofilm production	Number	Percentage
Strong biofilm producer	22	11.22
Moderate biofilm producer	58	29.59
Weak biofilm producer	91	46.43
Non-biofilm producer	25	12.76
Total	196	100.00

**Figure 3:**
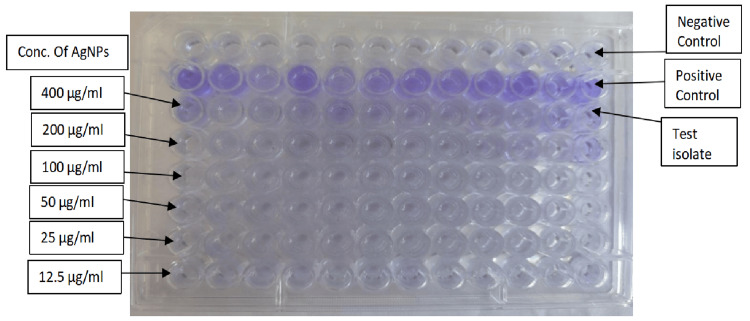
Antibiofilm assay: ELISA plate. ELISA: enzyme-linked immunosorbent assay; µg/mL: micrograms per milliliter; AgNPs: silver nanoparticles

A total of 80 isolates of moderate and strong biofilm producer categories were studied for an antibiofilm assay using AgNPs, according to Figure 3.

The best inhibitory effect was discovered with increasing concentration. Sixty-eight (85%) isolates were 60%-90% inhibited at 400 µg/mL NP concentration, as shown in Table 3. The chi-squared test was applied to two variables in the table, which are the range of percent inhibition and the number of isolates inhibited by AgNPs with increasing concentrations. The p-value is <0.0001, and the variables are significantly associated. The effective dose of biofilm inhibition was 50-400 µg/mL of inhibition.

**Table 3:** Antibiofilm effect of silver nanoparticles on biofilm-producing isolates (n = 80). µg/mL: micrograms per milliliter; χ^2^: chi square; DF: degree of freedom

Range of percent inhibition	Number of isolates inhibited by silver nanoparticles with increasing concentrations								
	12.5 µg/mL	25 µg/mL	50 µg/mL	100 µg/mL	200 µg/mL	400 µg/mL	χ^2^	DF	p-value
0%-30%	15 (18.75%)	7 (8.75%)	2 (2.5%)	1 (1.25%)	1 (1.25%)	1 (1.25%)	44.507	10	<0.0001
30%-60%	15 (18.75%)	21 (26.25%)	20 (25%)	15 (18.75%)	14 (17.5%)	11 (13.75%)			
60%-90%	50 (62.5%)	52 (65%)	58 (72.5%)	64 (80%)	65 (81.25%)	68 (85%)			
Total	80	80	80	80	80	80			

## Discussion

*P. aeruginosa* is a common bacterium that poses significant challenges in healthcare settings due to its multifactorial and exceptional antibiotic resistance. This resistance arises from both acquired and intrinsic mechanisms, such as the horizontal transfer of resistance genes, spontaneous mutations, and the production of enzymes like β-lactamases that degrade antibiotics, as well as biofilm production, efflux pumps that expel antibiotics, and low outer membrane permeability that limits drug entry and protects against treatment. This resistance makes therapy more difficult and contributes to its role as a major cause of HAIs, particularly in immunocompromised patients [11].

The increasing number of infections caused by MDR bacteria has become a significant threat in the medical world. This further exacerbates the challenge, underscoring the need for ongoing research and the advancement of new treatment strategies to address this significant public health threat. Multidrug resistance is due to the widespread usage of antibiotics. Multidrug resistance is a condition in which a microorganism is resistant to at least one antibiotic in three or more different groups of antibiotics. The microorganism is known to cause infections in many healthcare settings, especially in ICUs and surgical wards, and its antibiotic-resistant pattern varies across different regions [31].

There was a high prevalence of biofilm production in our study-tested isolates; 171 (87.24%) were biofilm producers by the TCP method. Similarly, high biofilm production was also noted in Iraq by Haji, who reported that out of 96, 84 (87.5%) isolates were biofilm producers by the TCP method in 2018 [26]. A study was conducted in Kancheepuram District, Tamil Nadu, India, by Swapna et al. in 2021 [32], in which, among a total of 87 isolates of *P. aeruginosa*, 68 (97%) were biofilm producers, and this finding was higher as compared with our study results. In contrast, others showed a lower rate of biofilm production. A study done by Shrestha in Nepal in 2019 [33] indicates that out of 90 isolates, only 29 (32.2%) were biofilm producers by the TCP assay. Another example is the survey carried out at Ambajogai, Maharashtra, by Kulkarni et al. in 2020 [34].

As per the criteria of Stepanović et al., biofilm production was grouped into four categories: strong, moderate, weak, and non-biofilm producers [35]. In our study, non-biofilm producers were 25 (12.76%). The biofilm producer category with a high occurrence was weak biofilm producers (91 (46.43%)), followed by moderate biofilm producers (58 (29.59%)) and strong biofilm producers (22 (11.22%)). Slightly similar results were observed in Brazil by Lima et al. in 2018, in which nine (22.5%) non-biofilm producers, 17 (42.5%) weak, 11 (27.5%) moderate, and three (7.5%) strong biofilm producers were observed [36]. Another study done in Iran by Kamali et al. observed that 67 out of 80 isolates (83.75%) were biofilm producers phenotypically; among them, 13 (16.25%) were strong, 27 (33.75%) moderate, and 27 (33.75%) weak; and 13 (16.25%) of the isolates were non-biofilm producers, comparable to our study [27]. A study from Egypt by Edward et al. reported biofilm formation (93 (89.4%)); among *P. aeruginosa* (n = 104), 54 (51.9%), 37 (35.5%), and 13 (12.5%) were weak, moderate, and strong biofilm producers, respectively [37].

In this study of biofilm producers, when tested for antibiofilm activity, 68 (85%) of isolates showed 60%-90% inhibition of biofilm production at 400 μg/mL AgNP concentration. A study by El-Telbany and El-Sharaki in 2021 [29] from Zagazig University, Egypt, found the highest reduction of *P. aeruginosa* biofilm when treated with 200 μg/mL of AgNPs, followed by 100 and 50 μg/mL in comparison with the control. This study shows that all AgNP concentrations were effective in the prevention of *P. aeruginosa* growth and in the destruction of their biofilms. Similarly, another study by Palanisamy et al. from Selangor, Malaysia, reported that the activity of AgNPs is highest at the concentration of 20 μg/mL, with an inhibition rate of 67%, and optimal at a bacterial concentration of 10^4^ cfu/mL [28]. Ebrahimi et al. from Iran reported that AgNPs had more than 90% inhibitory effect on biofilm formation [30]. Variations may be due to the size, shape, surface charge, and synthesis method of the NPs.

Limitations

Only one type of NP was used. Future studies may be done by using different NPs. Also, the study was in vitro, and in vivo trials to check NP tolerability are needed.

## Conclusions

*P. aeruginosa* is a significant hospital-associated pathogen, as indicated by its isolation rate of 16.15%, which emphasizes the need for focused initiatives to manage its impact in clinical settings. Most of the isolates, i.e., 87%, were biofilm producers making the isolates with higher resistance patterns. Nanotechnology-based strategies have shown promise in the battle against biofilms, providing several benefits over conventional antibacterial treatments. AgNPs can help improve public health by combating biofilms. By utilizing their special qualities and modes of action against microbial biofilms, NPs offer adaptable and promising methods for preventing biofilm formation.
